# Impact of Community-Oriented Medical Education on Medical Students’ Perceptions of Community Health Care: Qualitative Study

**DOI:** 10.2196/84406

**Published:** 2026-01-19

**Authors:** Kiyoshi Shikino, Kazuyo Yamauchi, Nobuyuki Araki, Naoto Ozaki, Yu Kamata, Shinya Aoki, Yota Katsuyama, Daichi Sogai, Mai Miyamoto, Kensuke Yoshimura, Takeshi Oki, Shoichi Ito

**Affiliations:** 1 Department of Community-Oriented Medical Education Graduate School of Medicine Chiba University Chiba Japan; 2 Department of Medical Education, Dokkyo Medical University Tochigi Japan; 3 Department of General Medicine Shioda Hospital Katsuura Japan; 4 Sanbunomori Clinic Sanmu Japan; 5 Department of General Medicine Sanmu Medical Center Sanmu Japan; 6 Department of Internal Medicine Isumi Medical Center Isumi Japan; 7 Center for Next Generation of Community Health Chiba University Hospital Chiba Japan; 8 Trinity Neurology Clinic Sakura Japan; 9 Department of Medical Education Graduate School of Medicine Chiba University Chiba Japan

**Keywords:** community health care, community-based medical education, community-oriented medical education, physician maldistribution, career perceptions, community hospital, community education, faculty development

## Abstract

**Background:**

Physician maldistribution remains a global challenge, with Japan’s rural regions facing critical health care shortages. Regional quota programs aim to attract medical students to underserved areas; however, their effectiveness in fostering long-term commitment is uncertain. Community-oriented medical education (COME) programs aim to address this issue by developing students’ understanding and dedication to rural health care.

**Objective:**

This study investigated the impact of an enhanced COME program, featuring increased early clinical exposure and faculty development, on first-year regional quota medical students’ perception of community health care at Chiba University.

**Methods:**

We conducted a cross-sectional qualitative study comparing 2 cohorts, 20 students enrolled from the existing COME course (April-December 2021) and 20 from the revised course (April-December 2022). The revised course included an additional day of community-based clinical exposure supervised by COME-trained attending physicians. Students’ written reflections were analyzed using qualitative content analysis and categorized according to the Fink Taxonomy of significant learning, comprising 6 domains, including foundational knowledge, application, integration, human dimension, caring, and learning how to learn. Reflections were synthesized into higher-order themes crosswalked to the Fink domains.

**Results:**

Demographics were similar between the 2021 and 2022 cohorts. In 2021, 311 learning codes were identified across foundational knowledge (n=128), application (n=91), integration (n=40), human dimension (n=16), caring (n=30), and learning how to learn (n=6). In 2022, codes increased to 385, with notable growth in caring (n=58) and human dimension (n=57), alongside increases in learning how to learn (n=15) and integration (n=45). Theme-based synthesis identified four overarching themes: (1) community health care as an interconnected, resource-constrained system; (2) patient-centered relationships and trust through communication and teamwork; (3) emerging professional identity and responsibility toward community service; and (4) developing a self-directed learning orientation for community practice. Qualitative analysis revealed that students gained a deeper understanding of patient-centered care, interprofessional collaboration, and social challenges in rural health care. The consistency in the foundational knowledge domain underscored a stable conceptual foundation, while the increase in affective and reflective domains reflected greater emphasis on interpersonal, value-oriented, and reflective learning in the revised cohort.

**Conclusions:**

Enhancements of the COME program, including additional early clinical exposure and faculty development, were associated with improved students’ perceptions of community health care. The increased focus on the caring and human dimension domains underscores the role of practical experiences in fostering collaboration, communication, and patient-centered care. The theme-based synthesis further suggests that the revised program prompted more frequent reflections on professional identity formation and self-directed learning while maintaining a stable foundation of community health care concepts. Mentorship by community hospital attendings, alongside structured clinical exposure, appears crucial in shaping medical students’ understanding and commitment to rural medicine. Ongoing longitudinal evaluations are warranted to assess the sustained impact of COME programs on career trajectories in underserved areas.

## Introduction

Physician maldistribution is a global concern affecting many nations [1-6]. In Japan, rural regions are facing a critical shortage of health care providers [7,8] due to urbanization [9-11]. To address this issue, numerous Japanese medical schools have introduced regional quota programs designed to motivate students to practice community or rural medicine [7,8,12]. These programs offer scholarships tied to service agreements in designated regions to address the scarcity of physicians. As of 2015, 70 out of 79 Japanese medical schools offered regional quota programs, enrolling 1541 students [7].

However, the effectiveness and ethical implications of these programs are debatable. Students assigned to regional quotas may become less inclined to work in their designated regions as they progress through their academic programs [8]. Therefore, it is essential to develop community-oriented medical education (COME) courses that not only impart knowledge but also cultivate students’ commitment to serving in underserved communities [13].

The Japanese government has high expectations for community-based medical education (CBME) and has made it a compulsory component of medical training [13-15]. Through direct participation in CBME, medical students and resident physicians can acquire practical skills, knowledge, and attitudes pertinent to clinical practice [16]. Nonetheless, questions remain regarding the long-term sustainability and impact of these programs, including student retention in rural areas [14]. Thus, continuous evaluation and enhancement are critical to effectively mitigate health care provider shortages in rural areas in Japan [8].

Herein, we aimed to investigate the educational impact of an enhanced COME program, which incorporated increased early clinical exposure and faculty development for community hospital attendings, on first-year regional quota medical students at Chiba University. Specifically, we explored students’ “career perceptions,” defined as their developing understanding of professional identity, motivation toward community service, and long-term orientation toward rural medical practice. We hypothesized that participation in the enhanced COME program would strengthen students’ sense of professional responsibility and commitment to working in underserved areas by deepening their reflection on real-world community health care experiences. In this study, the term “career perceptions” refers to students’ evolving understanding of their future professional roles, motivations, and attitudes toward community health care. These perceptions encompass not only students’ intentions to work in rural or underserved settings but also their sense of social responsibility, empathy, and professional identity as future physicians.

## Methods

### Study Design

A cross-sectional survey was conducted to assess the influence of a COME program, designed by the Chiba University School of Medicine, on medical students’ career perceptions. In this study, “career perceptions” were defined as students’ reflective understanding of their professional identity, motivation toward community service, and long-term orientation toward rural medical practice. The survey was designed to collect qualitative data from students’ written reflections rather than quantitative responses, allowing for an in-depth exploration of their perceptions and learning experiences. Qualitative research typically involves data in the form of words instead of numbers and is particularly appropriate for understanding participants’ experiences, thoughts, and motivations [17].

This study encompassed 2 cohorts, including 20 first-year regional quota medical students from April to December 2021, representing the existing course structure, and 20 students from April to December 2022, representing a revised course with additional early clinical exposure. Before participating in the COME program, the students had already completed the interprofessional education (IPE) step 1 course earlier in the academic year, which may have influenced their perceptions and learning outcomes in the COME program.

### Participants and Settings

Participants were first-year medical students enrolled in Chiba University under a regional quota system, participating in a year-long COME program. All participants were Japanese medical students enrolled in the regional quota program at Chiba University. The 2021 (n=20) and 2022 cohorts (n=20) each consisted of first-year students aged 18-24 years and 18-21 years, respectively. Sex assigned at birth (male or female) was collected as a demographic variable. This program included didactic sessions and practical experiences designed to enhance students’ understanding of and commitment to community health care.

### Selection Process for Regional Quota Students

Students applying to Chiba University School of Medicine must choose between applying under the general admission track or the regional quota track. Admission decisions are based on the scores from the common test for university admissions, a written examination, and an interview. Students admitted under the regional quota track are obligated to work within Chiba Prefecture for 9 years after graduation, fulfilling a service agreement. In return, they receive financial support through educational subsidies as an incentive. This system is intended to attract and retain health care professionals in underserved areas of the prefecture.

### COME at Chiba University

Chiba Prefecture is one of the regions in Japan with a significant disparity in the geographical distribution of physicians, creating an urgent need to ensure the retention of medical professionals in community health care settings. In response, the COME program was introduced to address this issue through targeted human resource development. The program aims to cultivate medical students’ understanding of and commitment to practicing in underserved areas, thereby contributing to sustainable health care solutions in the region.

### Facilities and Roles of Community Hospital Attending

In 2022, the COME program was implemented across 4 facilities, each staffed with attending physicians dedicated to community-based medical education. These community hospital attendings play a critical role in educating medical students and creating an optimal learning environment. Additionally, they participate in weekly collaborative faculty development sessions at Chiba University, integrating practical experience in community health care with theoretical frameworks, ensuring a comprehensive approach to medical education in underserved areas.

### IPE Step 1 Course

Before participating in the COME program, all students had completed the IPE Step 1 course, which establishes the necessary professional attitudes for health care practitioners and develops their ability to communicate effectively with patients, service users, and students from other faculties. At the end of the course, students are expected to reflect on the requirements for their professional growth, contribute effectively to team efforts, and respect interprofessional collaboration. These competencies likely influenced students’ perceptions and learning outcomes in the subsequent COME program.

### Enhancements to the COME Program

**Figure 1:**
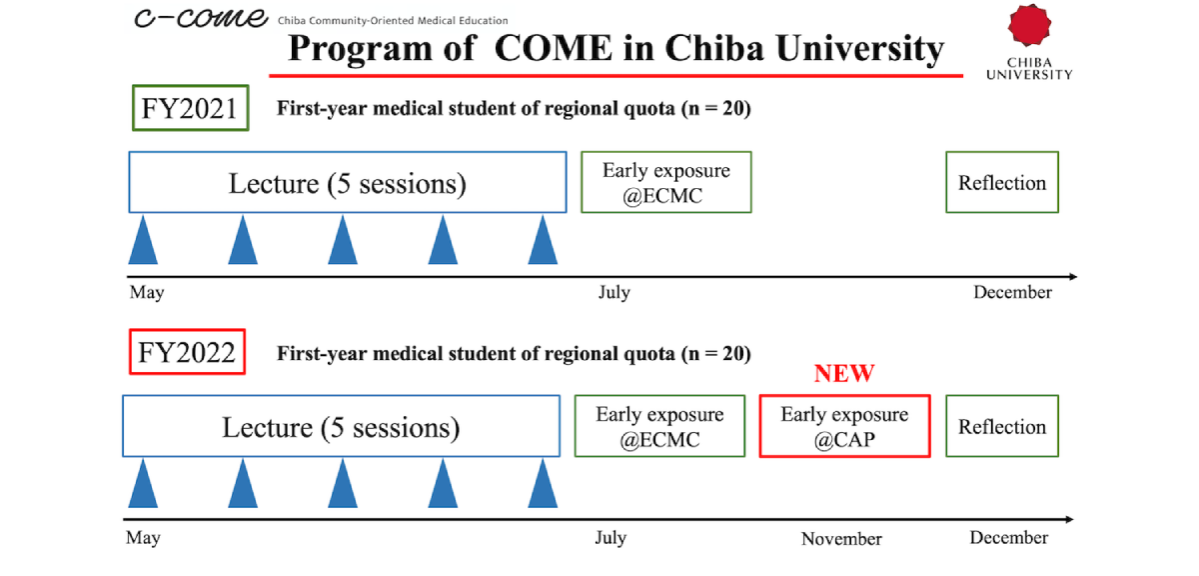
Structure and timeline of the community-oriented medical education (COME) program at Chiba University for first-year regional quota medical students in 2021 and 2022. CAP: community-oriented medical education–trained attending physicians; ECMC: Eastern Chiba Medical Center; FY: final year.

In 2021, the COME program consisted of five lecture sessions, one day of early clinical exposure, and subsequent reflection sessions (Figure 1). During this day, students mainly observed outpatient and inpatient activities at Eastern Chiba Medical Center, attended multidisciplinary team meetings, and learned about hospital functions and community health care delivery. Their participation was primarily observational. In 2022, the program was enhanced with an additional day of clinical exposure, orchestrated by COME-trained attending physicians serving as community preceptors. This additional day was implemented at community hospitals where students shadowed COME-trained attendings, interacted directly with patients, and discussed diagnostic reasoning and care planning. Students also engaged in reflective discussions with their mentors, receiving formative feedback on communication and professionalism. Early clinical exposure allowed students to learn directly from physicians who served as supervisors and educational leaders. Before clinical exposure, attending physicians engaged in targeted mentorship through personalized communication via email or online conferences to understand each student’s individual needs and learning objectives. The faculty development program, consisting of 40 sessions annually, equipped these physicians with pedagogical skills to provide personalized guidance throughout the medical education process. These attending physicians, strategically positioned in regions with physician shortages, underwent comprehensive faculty development. This development entailed 40 sessions annually, each lasting 2 hours, and covered various pedagogical topics, including educational technology, simulation teaching, and assessment methodologies. These sessions were part of a broader faculty development program to enhance the physicians’ overall educational capabilities and equip them to provide personalized guidance throughout the students’ medical education. Overall, the 2022 revision represented a shift from passive observation to active engagement and mentorship-based learning, which deepened students’ understanding of community health care and enhanced their sense of professional identity. These enhancements aimed to provide students with practical experiences and mentorship that deepen their understanding of community health care and address the challenges faced in underserved areas [18].

In Figure 1, it can be found that, in 2021, the program comprised 5 lecture sessions, one early clinical exposure at Eastern Chiba Medical Center (ECMC), and reflection activities. In 2022, the revised program added a new early clinical exposure component at hospitals staffed by COME-trained attending physicians (CAP), integrating faculty development initiatives to enhance community-based mentorship.

### Data Collection

#### Students’ Reports

A qualitative inquiry was conducted. To measure the educational impact, we analyzed students’ reports from 2021-2022 as qualitative data. These reports addressed 4 key questions regarding students’ understanding of community medicine, health care challenges, their envisioned role in local health care, and the competencies required for these roles (Multimedia Appendix 1).

#### The Fink Taxonomy of Significant Learning

**Figure 2:**
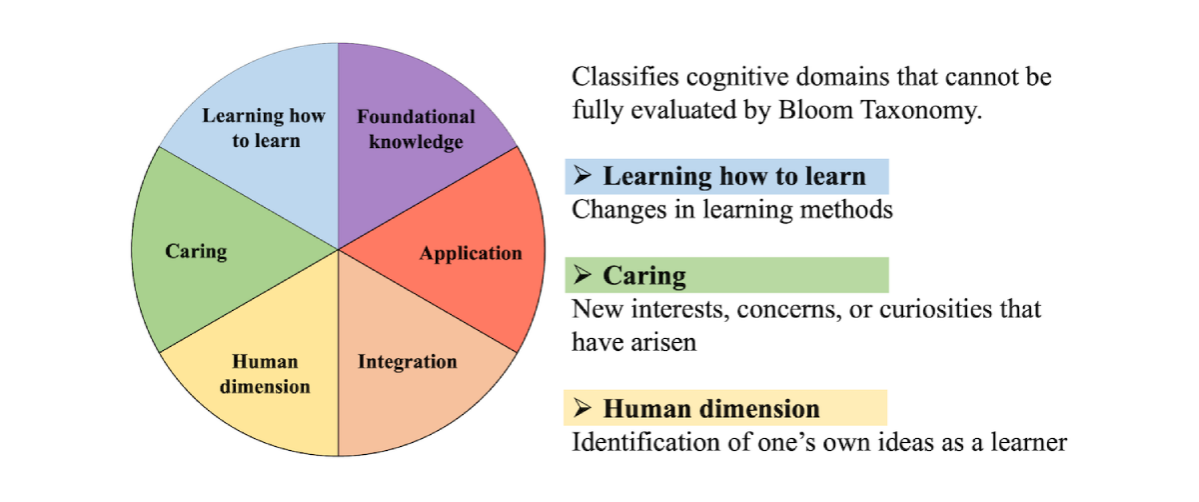
Framework of the Fink Taxonomy of significant learning used for qualitative content analysis.

The Fink Taxonomy [19] is distinct in its categorization of cognitive domains, offering a broader scope than the Bloom Taxonomy [12,20-22]. It emphasizes the “Human dimension”–students’ self-awareness as learners; “Caring” the development of new interests and concerns; and “Learning how to learn” the adaptation and improvement of students’ learning approaches (Figure 2 and Multimedia Appendix 2) [19].

In Figure 2, the taxonomy classifies learning outcomes into 6 interrelated domains, foundational knowledge, application, integration, human dimension, caring, and learning how to learn. This framework guided the categorization of students’ written reflections to evaluate changes in cognitive, affective, and interpersonal learning domains beyond what is captured by Bloom Taxonomy.

### Data Analysis

#### Content Analysis

Content analysis was conducted using the Fink Taxonomy of significant learning to evaluate students’ perceptions of community health care. This taxonomy categorizes cognitive process dimensions into various categories and subcategories [23,24]. KS independently read and coded all transcripts. Subsequently, the descriptors were discussed, identified, and agreed upon by KS, K Yamauchi, and NA. Similar codes were then grouped into categories and subcategories as they emerged from the data. These categories and subcategories were regularly reviewed and discussed by authors experienced in qualitative research to ensure the credibility of the findings [25]. Data saturation was assessed through an iterative process of constant comparison. After each round of coding, the research team reviewed whether new concepts or categories emerged. When no additional themes or codes were identified across 3 consecutive transcripts, we considered both thematic and code saturation to have been achieved [26,27]. The authors discussed and reached a consensus on all further changes, achieving code saturation. We adopted an inductive and deductive content analysis approach to balance theory-driven and data-driven coding. Initially, inductive coding allowed themes to emerge naturally from the students’ reflections, ensuring that novel insights were not constrained by pre-existing frameworks. Subsequently, deductive categorization was applied using the Fink Taxonomy to systematically organize the emergent themes into recognized dimensions of significant learning. This combined approach enhanced the analytical rigor and minimized the risk of forcing data into preset categories [22].

Concepts for each cognitive process dimension in the Fink Taxonomy [20] were analyzed, and the number of units of analysis for each concept was counted. The researchers then grouped similar codes into themes and checked to see which dimension of the cognitive process they corresponded to.

#### AI-Assisted Analytic Support (Generative AI)

Additionally, to enhance the rigor and transparency of our qualitative analysis, we complemented the human coding process with a generative artificial intelligence (AI) tool (ChatGPT 5; OpenAI) [25,26]. The AI tool was prompted to identify potential thematic clusters and semantic relationships within anonymized textual data, following best practices for AI-assisted qualitative research as outlined in recent literature [28-31]. Specifically, the AI was used to (1) propose candidate thematic clusters and relationships across codes/excerpts, (2) suggest alternative categorizations and labels, and (3) identify potential negative cases or boundary instances that did not fit preliminary themes. Only deidentified materials (eg, anonymized excerpts and/or code lists) were entered into the tool; no personal identifiers were provided. The AI outputs were critically reviewed, refined, and confirmed through team discussion to prevent model-induced bias. AI outputs were treated as hypotheses for discussion rather than definitive findings, and the research team documented whether each suggestion was adopted, refined, or rejected after checking against the original data. This triangulated approach improved coding consistency while ensuring that all final interpretations and coding decisions were made exclusively by human researchers [29,31,32]. Although formal interrater reliability statistics were not calculated, coding credibility was ensured through iterative peer review, team consensus discussions, and triangulation using AI-assisted content verification. Because we did not systematically archive full prompt-response transcripts during the earlier analytic iterations, we provide representative example prompts (and abbreviated outputs) in Multimedia Appendix 3 to enhance transparency and reproducibility.

#### Reflexivity

We conducted a qualitative study using content analysis to determine the effectiveness of the newly developed program in 2022 on students’ perceptions of community health care. In qualitative research, researchers’ backgrounds can significantly influence data interpretation and the construction of findings and conclusions, necessitating careful reflexivity. Reflexivity was used to account for researchers’ potential biases.

In this study, all authors were clinical educators with experience in COME, providing the necessary expertise to understand and interpret the data accurately. The authors were trained in educational theory (KS, K Yamauchi, and SI) and primary care (SA, Y Kamata, DS, MM, and TO), enabling them to approach the analysis from a well-rounded perspective. This dual expertise ensured a comprehensive understanding of the educational and clinical aspects of community-oriented medical education programs. This reflective approach aimed to ensure the credibility and reliability of our findings and provide valuable insights into the factors contributing to successful training programs.

### Ethical Considerations

This study was approved by the Ethics Review Committee of the Graduate School of Medicine, Chiba University (Approval No. 3425, approved on October 4, 2019). All work involving research on human participants complied with the Declaration of Helsinki. The procedures for obtaining informed consent were explained to the medical students, who were also informed that this study would not affect their grades. The researchers explained to the participants and obtained their informed and voluntary consent. No financial or material compensation was provided to participants for their involvement in this study.

## Results

Content analysis of the reports from first-year regional quota medical students revealed distinct patterns across the academic years 2021 and 2022, categorized according to the Fink Taxonomy of significant learning (Tables 1 and 2). The demographic characteristics of participants were similar between the 2021 and 2022 cohorts. The median age was 19 years in both groups (2021: 18-24 years; 2022: 18-21 years), and the distribution of sex assigned at birth did not differ significantly (2021: 75% male, 25% female; 2022: 70% male, 30% female; *P*>.99).

**Table 1:** Results of qualitative content analysis of first-year regional quota medical students’ reflection reports in the 2021 community-oriented medical education (COME) program at Chiba University (N=20), crosswalked to the Fink Taxonomy of significant learning.

Theme, key synthesis, and crosswalk to the Fink domains				Subcategories (code counts)
**Theme 1: community health care as an interconnected, resource-constrained system**				
	**Community health care is understood as a local system shaped by needs, constraints, and intersector collaboration.**			
		Primary domains: Foundational knowledge	Specific initiatives (n=48), challenges/current state (n=38), definition/role (n=26), aging (n=16)	
		Secondary domains: Integration	Challenges/responses (n=13), improvement of knowledge and skills (n=11), communication/cooperation (n=9), collaboration with community (n=7)	
**Theme 2:** **patient-centered relationships and trust through communication and teamwork**				
	**Communication is positioned as the practical foundation of patient-centered care and trust, supported by collaboration.**			
		Primary domains: Application	Communication and trust building (n=15)	
		Secondary domains: Human dimension Caring	Patient-centered health care and communication (n=6) Patient-centered health care (n=7), communication and collaboration (n=5)	
**Theme 3: emerging professional identity and responsibility toward community service**				
	**Students begin to articulate who they should become as physicians and how they should contribute to the community.**			
		Primary domains: Human dimension Caring	Patient-centered health care and communication (n=6), understanding and importance of community health care (n=5), general practice and broad knowledge (n=3), realization of the community health care field (n=2) Contribution and involvement in community health care (n=9), personal growth and learning (n=8), patient-centered health care (n=7), communication and collaboration (n=5), personal motivation and awareness (n=1)	
		Secondary domains: Application	Improve knowledge/skills (n=20), practice/problem-solving (n=18)	
**Theme 4: developing a self-directed learning orientation for community practice**				
	**Students emphasize experiential learning and continuous improvement as preparation for community practice.**			
		Primary domains: Learning how to learn	Practical experience and activity participation (n=2), improvement of skills and knowledge (n=2), improvement of communication skills (n=1), continuous learning and understanding of community (n=1)	
		Secondary domains: Application Foundational knowledge	Improvement of knowledge and skills (n=20), practice and problem-solving in community health care (n=18), communication and trust building (n=15), necessity and outlook of community health care (n=13), improvement of prevention and health awareness (n=9), use of information and communication technology (n=7), environmental preparation and collaboration in community health care (n=5), general practitioners and multidisciplinary knowledge (n=4) Specific initiatives in community health care (n=48), challenges and current state of community health care (n=38), definition and role of community health care (n=26), aging population and community health care (n=16)	

**Table 2:** Results of qualitative content analysis of first-year regional quota medical students’ reflection reports in the revised 2022 community-oriented medical education (COME) program at Chiba University (n=20), crosswalked to the Fink Taxonomy of significant learning.

Theme, key synthesis, and crosswalk to the Fink domains				Subcategories (code counts)
**Theme 1: community health care as an interconnected, resource-constrained system**				
	**Community health care is understood as a local system shaped by needs, constraints, and intersector collaboration.**			
		Primary domains: Foundational knowledge	Quality and supply of health care (n=51), shortage of physicians and medical resources (n=41), importance of communication and consultation (n=23), aging population and the community health care (n=13)	
		Secondary domain: Integration	Medical resources and collaboration (n=15), sustainability and technology in health care (n=12), patient-centered care method (n=10)	
**Theme 2:** **patient-centered relationships and trust through communication and teamwork**				
	**Communication is positioned as the practical foundation of patient-centered care and trust, supported by collaboration.**			
		Primary domains: Human dimension Application	Patient-centered health care and communication (n=26) Communication and trust building (n=15)	
		Secondary domain: Caring	Communication and collaboration (n=15), patient-centered health care (n=11)	
**Theme 3: emerging professional identity and responsibility toward community service**				
	**Students begin to articulate who they should become as physicians and how they should contribute to the community.**			
		Primary domains: Human dimension Caring	Challenges and improvements in community health care (n=21), importance and current state of community health care (n=10) Contribution and involvement in community health care (n=12), personal motivation and awareness (n=10), personal growth and learning (n=10)	
		Secondary domains: Integration	Ethics and communication (n=8)	
**Theme 4: developing a self-directed learning orientation for community practice**				
	**Students emphasize experiential learning and continuous improvement as preparation for community practice.**			
		Primary domains: Learning how to learn	Acquisition of broad knowledge (n=5), activities to broaden knowledge (n=4), interest and training (n=3), mandatory community health care training (n=2), improvement of communication skills (n=1)	
		Secondary domains: Application	Improvement of medical knowledge and skills (n=20), practice and problem-solving in community health care (n=20), community healthcare policy and support (n=8), executing ability and motivation (n=8), importance of community health care (n=6), use of information and communication technology (n=5)	

Across both cohorts, we identified four higher-order themes that synthesize students’ reflections and crosswalk to multiple domains of the Fink Taxonomy: (1) community health care as an interconnected, resource-constrained system; (2) patient-centered relationships and trust through communication and teamwork; (3) emerging professional identity and responsibility toward community service; and (4) developing a self-directed learning orientation for community practice (Tables 1 and 2; representative quotes are provided in Multimedia Appendices 4 and 5). While foundational understanding of community health care was evident in both years, the 2022 cohort more frequently expressed reflections aligned with affective and interpersonal learning (caring and human dimension) and reflective learning (learning how to learn), consistent with a more integrated pattern of “significant learning” in the Fink framework.

In Table 1, reports were analyzed using the Fink Taxonomy of significant learning, which categorizes learning into six domains. Subcategories and code counts are presented to support the theme-based synthesis and the crosswalk to the Fink domains. Representative quotes supporting each theme and subcategory are provided in Multimedia Appendix 4.

In Table 2, reports were analyzed using the Fink Taxonomy of significant learning, which categorizes learning into 6 domains. Subcategories and code counts are presented to support the theme-based synthesis and the crosswalk to the Fink domains. Representative quotes supporting each theme and subcategory are provided in Multimedia Appendix 5.

For the academic year 2021, 311 codes were identified. In thematic terms, the 2021 reflections predominantly emphasized building a baseline understanding of community health care systems and challenges (theme 1; foundational knowledge and integration), accompanied by early attention to communication as a core clinical practice (theme 2; application), and emerging awareness of professional roles and values (theme 3; human dimension and caring). In the learning how to learn category, 6 codes were noted, including practical experience and participation in activities (n=2), improvement of skills and knowledge (n=2), improvement of communication skills (n=1), and continuous learning and understanding of the community (n=1). Students frequently reflected on the value of experiential learning, with one noting that “Practical experience enhances learning,” emphasizing how direct participation reinforced their understanding of classroom content. The caring category included 30 codes, highlighting areas such as contribution to and involvement in community health care (n=9), personal growth and learning (n=8), patient-centered health care (n=7), communication and collaboration (n=5), and personal motivation and awareness (n=1). The human dimension category had 16 codes, emphasizing patient-centered health care and communication (n=6), understanding and importance of community health care (n=5), general practice and broad knowledge (n=3), and realization of the community health care field (n=2). The integration category comprised 40 codes, addressing challenges and responses in community health care (n=13), improvement of knowledge and skills (n=11), communication and cooperation (n=9), and collaboration with the community (n=7). The application category contained 91 codes, focusing on improvement of knowledge and skills (n=20), practice and problem-solving in community health care (n=18), communication and trust-building (n=15), necessity and outlook of community health care (n=13), improvement of prevention and health awareness (n=9), use of information and communication technology (n=7), environmental preparation and collaboration in community health care (n=5), and general practitioners and multidisciplinary knowledge (n=4). Finally, the foundational knowledge category included 128 codes, covering specific initiatives in community health care (n=48), challenges and the current state of community health care (n=38), the definition and role of community health care (n=26), and the aging population and community health care (n=16). One student reflected, “Community healthcare is necessary for supporting aging populations,” illustrating growing awareness of the social challenges faced by Japan’s communities.

In comparison, the academic year 2022 had 385 codes. The learning how to learn category expanded to 15 codes, covering the acquisition of broad knowledge (n=5), activities to broaden knowledge (n=4), interest and training (n=3), mandatory community health care training (n=2), and improvement of communication skills (n=1). The caring category rose significantly to 58 codes, with emphasis on communication and collaboration (n=15), contribution and involvement in community health care (n=12), patient-centered health care (n=11), personal growth and learning (n=10), and personal motivation and awareness (n=10). Students’ reflections often highlighted a stronger sense of teamwork and responsibility, exemplified by one participant who stated, “Collaboration among healthcare providers enhances communication and patient care.” Another remarked, “Personal motivation and awareness are key drivers for effective healthcare delivery,” underscoring the affective learning fostered by the revised program. The human dimension category also increased to 57 codes, focusing on patient-centered health care and communication (n=26), challenges and improvements in community health care (n=21), and the importance and current state of community health care (n=10). The integration category contained 45 codes, addressing medical resources and collaboration (n=15), sustainability and technology in health care (n=12), patient-centered care methods (n=10), and ethics and communication (n=8). The application category contained 82 codes, emphasizing improvement of medical knowledge and skills (n=20), practice and problem-solving in community health care (n=20), communication and trust-building (n=15), community health care policy and support (n=8), execution ability and motivation (n=8), importance of community health care (n=6), and use of information and communication technology (n=5). Finally, the foundational knowledge category remained consistent with 128 codes, focusing on the quality and supply of health care (n=51), shortage of physicians and medical resources (n=41), importance of communication and consultation (n=23), and aging population and community health care (n=13).

Comparing the data from 2 years, a notable increase was observed in the total number of codes, particularly in the caring and human dimension categories in 2022. Rather than relying solely on descriptive counts, the theme-based synthesis suggests a shift in emphasis from predominantly system- and knowledge-oriented descriptions (theme 1) toward reflections that more frequently articulated professional identity, values, and relationships in care (themes 2-3), alongside clearer intentions for continued learning (theme 4). These patterns are consistent with a more integrated profile across the Fink domains in 2022, while maintaining a stable baseline of foundational knowledge.

Beyond the individual code counts, several higher-level patterns emerged across both cohorts. First, the number of foundational knowledge codes remained stable across the 2 years, indicating a consistent baseline of understanding of community health care concepts. Second, the revised 2022 program demonstrated a marked increase in codes within the caring and human dimension categories. Third, learning how to learn codes also increased in 2022. Taken together, the thematic synthesis (Tables 1 and 2) indicates that the 2022 cohort’s reflections more often combined system understanding with interpersonal, value-oriented, and reflective learning elements, whereas the 2021 cohort’s reflections were more concentrated in foundational and application-oriented descriptions.

## Discussion

### Summary of Key Findings

This study aimed to evaluate the educational impact of a COME program on first-year regional quota medical students at Chiba University. Through qualitative content analysis based on the Fink Taxonomy of significant learning, we identified substantial changes in students’ perceptions and reflections following the program revision. The revised COME course, which incorporated additional early clinical exposure and faculty development for community hospital attendings, emphasized affective and interpersonal learning, as well as reflective learning—particularly within the caring, human dimension, and learning how to learn domains—while maintaining a stable baseline of foundational knowledge. These shifts were most apparent in themes 2-4, which foreground patient-centered relationships, emerging professional identity, and self-directed learning orientation. This aligns with the study’s objective—to explore how early community-based experiences influence students’ understanding of and commitment to community health care—and indicates that experiential learning opportunities in real clinical contexts contribute meaningfully to professional identity formation among first-year medical students.

### Interpretation and Comparison With Previous Studies

Across both cohorts, several higher-level patterns emerged, offering broader insight into the educational impact of the revised COME program. While the number of foundational knowledge codes remained stable, indicating a consistent baseline understanding of community health care concepts, the revised 2022 program produced substantial increases in the caring, human dimension, and learning how to learn categories. Importantly, beyond these category-level changes, the theme-based synthesis (themes 1-4) indicates that students’ reflections in 2022 more often integrated system understanding with interpersonal, value-oriented, and reflective elements of learning (themes 2-4). These findings suggest that the experiential and interpersonal elements of the program contributed to deeper affective, interpersonal, and reflective learning, extending beyond the acquisition of foundational knowledge.

### Learning How to Learn and Lifelong Learning

The expansion of the learning how to learn category from 6 codes in 2021 to 15 in 2022 highlights the development of students’ reflective and self-directed learning capacities.

This pattern aligns with theme 4 (developing a self-directed learning orientation for community practice), in which students increasingly recognized the importance of continuous learning and professional growth, as reflected in comments such as “Acquiring a broad range of knowledge is important for comprehensive healthcare” and “Ongoing interest and training are essential for professional development.” This finding resonates with previous studies emphasizing the importance of reflective learning and self-regulation in developing lifelong learning competencies [32-34]. The structured faculty mentorship and feedback embedded in the revised program may have contributed to this growth by encouraging students to articulate their learning needs and apply theoretical knowledge to real-world contexts.

### Caring and Human Dimension: Growth in Empathy and Collaboration

A major finding was the substantial increase in the caring (from 30 to 58 codes) and human dimension (from 16 to 57 codes) categories. These domains were central to themes 2 and 3, which foregrounded patient-centered relationships, teamwork, and emerging professional identity and responsibility toward community service. Students’ reflections increasingly addressed interpersonal communication, collaboration, and patient-centered care. One student stated, “Collaboration among healthcare providers enhances communication and patient care,” while another reflected, “Patient-centered healthcare ensures that the care provided meets the needs of the patient.” These reflections demonstrate an affective shift toward empathy and teamwork, suggesting that early exposure to real patient encounters and mentorship in community hospitals fostered students’ understanding of the relational aspects of medicine. This aligns with prior research demonstrating that community-based and interprofessional learning environments enhance empathy and communication among medical students [35-37]. Similarly, our findings echo studies emphasizing that authentic, longitudinal clinical experiences—particularly those in underserved settings—help shape professional identity and strengthen social accountability [38-40]. One student further noted, “Addressing the challenges in community healthcare requires continuous improvement efforts,” capturing a sense of responsibility and ongoing self-improvement that mirrors the iterative nature of clinical reasoning and patient care.

### Integration and Application: Connecting Theory to Practice

The integration category increased modestly (40 to 45 codes), while the application category slightly decreased (91 to 82 codes). In the theme-based synthesis, integration was the most visible in theme 1 (community health care as a system) and theme 3 (professional identity and responsibility), where students linked collaboration, sustainability/technology, patient-centered care methods, and ethics. For instance, a student commented, “Collaboration between medical institutions is essential for optimizing resources,” illustrating recognition of systemic and organizational aspects of health care delivery. However, the slight decline in application codes suggests that opportunities for practical problem-solving and leadership in community health planning could be expanded. Despite this, students continued to value applied knowledge, as shown in reflections such as, “Healthcare providers must be adept at problem-solving to address diverse community health issues.” This pattern implies that while the program successfully deepened conceptual and interpersonal understanding, future iterations could further reinforce practical application and leadership in community contexts. These insights are consistent with previous reports that emphasize the need for a balance between affective learning and skill-based experiential education [41-43].

### Foundational Knowledge: Consolidating Core Understanding

The foundational knowledge category remained stable with 128 codes in both years, reflecting consistent comprehension of fundamental community health care principles. This stable foundation corresponded to theme 1 in both cohorts, where students continued to demonstrate awareness of the structural challenges facing Japan’s health care system, particularly physician shortages and the aging population. Statements such as “Ensuring high-quality healthcare is critical for meeting community needs” and “Many rural areas face significant shortages of doctors and medical resources” highlight this awareness. This stability suggests that students entered the program with a basic understanding of community health needs and retained this foundation while expanding their capacity for empathy, reflection, and teamwork through experiential learning [44,45].

### Comparison With Existing Educational Models

The differences observed between the COME program and early clinical exposure within university hospitals further highlight the program’s unique contribution. Unlike hospital-based experiences that often emphasize specialized and controlled environments, the COME program situates students in authentic community settings where resource limitations and multidisciplinary collaboration are inherent. Students reflected that learning from community physicians provided unique insights into the realities of rural health care. As one student expressed,

Learning from physicians embedded in the community provided valuable insights into the challenges and rewards of rural healthcare.

This finding aligns with prior literature suggesting that early, community-facing experiences cultivate adaptability, resource awareness, and social accountability [46,47]. Furthermore, recent evidence indicates that such community-facing clinical exposure not only enhances engagement and learning outcomes but also fosters the development of professional identity, social accountability, and collaborative competencies essential for clinical reasoning [46-48]. In addition, context-specific curriculum design is crucial for optimizing the educational impact of such programs. Understanding regional variations in students’ study habits, learning preferences, and access to educational resources allows educators to adapt teaching methods—such as incorporating imaging-based learning modules, interprofessional collaboration, or problem-based approaches—to enhance engagement and relevance in local settings [49]. These insights highlight that while COME provides authentic, real-world exposure to health care challenges, tailoring curricular content and pedagogy to local educational contexts can further enhance learning outcomes and promote sustained motivation among medical students. By engaging with real patients and health care teams early in their education, students not only improved their communication skills but also developed a sense of mission toward addressing regional health disparities.

### Limitations

This study has some limitations. First, the sample size was limited to first-year regional quota medical students at a single institution (n=40), which restricts the generalizability of the findings. The participants were from a single admission track; hence, demographic and baseline characteristics between the 2021 and 2022 cohorts were not formally analyzed, which may introduce selection bias. However, both cohorts had similar demographic profiles, showing comparable age ranges and distributions of sex assigned at birth. In addition, the results were derived from students’ written reflections rather than objective performance-based assessments. Therefore, while these reflections provide valuable insights into students’ perceptions and learning processes, they do not directly capture behavioral or performance-level outcomes. The study also relied on self-reported data, which may be subject to bias. The qualitative nature of the content analysis may also limit the reproducibility of the results, as it involves subjective data interpretation. Furthermore, the study used a cross-sectional design comparing 2 adjacent cohorts without a control group; thus, the observed improvements might reflect cohort effects, prior IPE exposure, or faculty-related factors rather than the revised COME program itself. The analysis also relied primarily on one coder, without interrater reliability testing, audit trails, or member checking, which could affect analytical rigor. In addition, applying the Fink Taxonomy a priori may have constrained data interpretation by fitting responses into predefined categories. However, previous qualitative studies have suggested that increases in code frequency and diversity—when analyzed within a validated theoretical framework—can indicate broader and deeper levels of learning and reflection [23,24]. Recent evidence also supports this interpretation, showing that analyses grounded in theoretically informed frameworks such as the Fink Taxonomy can meaningfully capture students’ cognitive and affective development through code expansion and thematic diversification [50]. Therefore, in this study, the observed growth in the number and variety of codes (eg, “Caring,” “Human dimension”) was interpreted as a qualitative indicator of students’ multidimensional learning development rather than a purely quantitative change. Nonetheless, no objective or longitudinal outcomes (eg, rural practice retention or sustained career engagement) were assessed. Another limitation is the short duration of the study, which spanned only 2 academic years; longer-term studies are needed to evaluate the sustained impact of the program on students’ career choices and retention in rural health care settings. Finally, the study did not account for external factors that may have influenced students’ perceptions, such as their personal experiences, socioeconomic backgrounds, or individual learning preferences. Tailoring educational strategies to align with students’ learning styles and real-world experiential opportunities may foster more effective and personalized medical education, ultimately enhancing both theoretical understanding and practical competency [51]. Future research should incorporate such individualized approaches and explore how these factors interact with community-based learning outcomes.

### Conclusions

Enhancements to the COME program at Chiba University, including early clinical exposure and the involvement of faculty-development-trained community hospital attendings, significantly improved students’ understanding of patient-centered and community-oriented health care. Our theme-based synthesis suggests that the revised program was associated with greater emphasis on patient-centered relationships, emerging professional identity, and self-directed learning (themes 2-4), while maintaining a stable baseline of foundational understanding (theme 1). These experiences cultivated empathy, reflective learning, and communication skills, which are essential for generalist physicians serving in underserved areas. Beyond these educational benefits, the findings have broader implications for medical education policy and workforce distribution. Structured community-based programs such as COME may contribute to addressing Japan’s rural physician shortage by fostering students’ identification with community health care roles early in their training. Continual evaluation and iterative refinement of COME programs are necessary to ensure that they remain responsive to regional health care needs and effectively cultivate physicians who are competent, compassionate, and socially accountable.

## Appendix

Multimedia Appendix 1 Students’ reports.

Multimedia Appendix 2 The Fink Taxonomy of significant learning.

Multimedia Appendix 3 Representative prompts and outputs for artificial intelligence–assisted qualitative content analysis.

Multimedia Appendix 4 Results of qualitative content analysis of first-year regional quota medical students’ reflection reports in the 2021 community-oriented medical education program at Chiba University (n=20).

Multimedia Appendix 5 Results of qualitative content analysis of first-year regional quota medical students’ reflection reports in the revised 2022 community-oriented medical education program at Chiba University (N=20).

## Abbreviations

AI: artificial intelligence
CBME: community-based medical education
COME: community-oriented medical education
IPE: interprofessional education

## Data Availability

The dataset generated or analyzed during this study is available from the corresponding author on reasonable request.
