# Supplementary material for: Impact of Community-Oriented Medical Education on Medical Students’ Perceptions of Community Health Care: Qualitative Study
Source: JMIR Med Educ. 2026 Jan 19;12:e84406. doi: 10.2196/84406 (PMC12865343; doi:10.2196/84406)
Supplement: Multimedia Appendix 1 [file mededu_v12i1e84406_app1.docx]

**Supplementary file 1. Students’ Reports**

**[Assignment 1]**

Describe the learning outcomes you have achieved through lectures, practical training, and self-directed learning in the course "Community Medicine" in 400–800 words.

**[Assignment 2]**

Based on the learning outcomes from the lectures, practical training, and self-directed learning in the course "Community Medicine," write a report of 800–1200 words that includes the following points:

1. What is community medicine?
2. What challenges need to be addressed in community medicine in Chiba Prefecture?
3. What role do you envision playing in the community medicine of Chiba Prefecture?
4. What qualities and skills must you acquire to fulfill the above role?

Note: Do not use generative AI to create the report.
