# Supplementary material for: Impact of Community-Oriented Medical Education on Medical Students’ Perceptions of Community Health Care: Qualitative Study
Source: JMIR Med Educ. 2026 Jan 19;12:e84406. doi: 10.2196/84406 (PMC12865343; doi:10.2196/84406)
Supplement: Multimedia Appendix 2 [file mededu_v12i1e84406_app2.docx]

**Supplementary file 2. Fink’s Taxonomy of Significant Learning**

| **Category of Learning** | **Meaning** |
| --- | --- |
| **Learning How to Learn** | Students will develop the ability to learn better (more efficiently and effectively), both in this course and in life in general. |
| **Caring** | Students will care more deeply about this subject or issues related to this subject. |
| **Human Dimension** | Self: Students will better understand themselves. |
|  | Others: Students will be able to interact positively and productively with others. |
| **Integration** | Students will be able to identify the relationship between “x” and “y”. |
| **Application** | Students will know how to “do” important tasks. |
| **Foundational Knowledge** | Students will understand and remember key concepts, terms, relationships, facts, etc. |
