# Supplementary material for: Impact of Community-Oriented Medical Education on Medical Students’ Perceptions of Community Health Care: Qualitative Study
Source: JMIR Med Educ. 2026 Jan 19;12:e84406. doi: 10.2196/84406 (PMC12865343; doi:10.2196/84406)
Supplement: Multimedia Appendix 3 [file mededu_v12i1e84406_app3.docx]

**Supplementary file 3. Representative prompts and outputs for AI-assisted qualitative content analysis**

## **Overview**

Generative AI (ChatGPT 5, OpenAI) was used as an assistive analytic aid to support qualitative content analysis. AI outputs were treated as hypotheses for team discussion rather than findings. All final coding decisions, theme definitions, and interpretations were made exclusively by the human research team.

### *Data provided to the AI tool*

### Only **de-identified** materials were entered (eg, anonymized excerpts and/or code lists).

### No personal identifiers (such as names, specific locations, or dates of birth) were included.

### *How AI outputs were used*

AI was used to:

1. propose candidate thematic clusters and semantic relationships across codes/excerpts,
2. suggest alternative categorizations/labels, and
3. identify potential negative cases or boundary instances.
   The research team reviewed each output against the original data and documented whether suggestions were adopted, refined, or rejected.

### *Note on transcript availability*

### Full prompt–response transcripts were not systematically archived during early analytic iterations. This appendix provides representative prompts (and abbreviated outputs) to enhance transparency and reproducibility.

## **Prompt Set 1: Framework mapping to Fink’s Taxonomy (deductive coding)**

### *Prompt 1 (Framework mapping; single best-fit)*

**Prompt:**
You are assisting qualitative content analysis. Use only the provided de-identified excerpts/codes. Do not add information that is not present in the data. If insufficient, respond ‘Unclear’ and explain why. Using Fink’s Taxonomy of Significant Learning with the following dimensions and study-tailored operational definitions, assign **one best-fitting** dimension to each meaning unit/excerpt below. If none of the options fit, label it ‘Outside framework’.

- Foundational Knowledge: understanding of community healthcare, local health systems, roles of facilities/professions, or healthcare challenges (factual/conceptual learning).
- Application: applying knowledge/skills in practice or problem-solving, including clinical reasoning, communication approaches, or care planning in a community setting.
- Integration: connecting ideas across concepts/experiences/disciplines; linking patient–family–team–community resource perspectives.
- Human Dimension: self-awareness, professional identity formation, perceived roles/responsibilities as a future physician, understanding others (patients/families/team).
- Caring: changes in values/empathy/motivation/commitment toward community service or underserved areas.
- Learning How to Learn: reflection/metacognition; recognizing learning needs; strategies for self-directed learning; intentions for continued growth.

Output format: Unit ID | Assigned dimension | Justification citing key phrases (verbatim). Units: [paste de-identified meaning units/excerpts].

**Abbreviated output (example format):**

- U03 | Human Dimension | “I realized what kind of doctor I want to become…”
- U07 | Integration | “Connecting outpatient care with community resources…”
- U12 | Learning How to Learn | “I need to study X; I will use Y method…”

**How the team used this output:**

- Used as a consistency check for deductive categorization; final assignments confirmed/modified by team discussion.

## **Prompt Set 2: Thematic synthesis/clustering (higher-order themes)**

### *Prompt 2 (Candidate theme generation)*

**Prompt:**
Using only the de-identified data below, propose **3–5 candidate higher-order themes** that synthesize the reflections. For each theme provide: (a) theme label, (b) 1–2 sentence definition, (c) included excerpt IDs (or codes), and (d) 2 supporting excerpts/phrases (verbatim). Then propose **one alternative clustering solution** (different grouping) to challenge the first solution. Data: [paste excerpt IDs + excerpts] and/or [paste code list + short definitions].

**Abbreviated output (example format):**

- Theme 1 (label): …

Definition: …

Included IDs: U01, U05, U09…

Supporting phrases: “…” / “…”

- Alternative clustering: (briefly list how themes would differ)

**How the team used this output:**

- Treated as candidate thematic structures; compared with human-generated themes; adopted/refined/rejected based on fit with original excerpts.

## **Prompt Set 3: Negative case / boundary case search (rigor)**

### *Prompt 3 (Negative/boundary cases)*

### **Prompt:** Here are the current themes and definitions: [paste themes]. Identify any excerpts that do **not fit well** into these themes (negative cases or boundary instances). For each, explain why it conflicts and suggest how to refine theme boundaries (revise definitions, split/merge themes) **without forcing the data**. Data: [paste excerpt IDs + excerpts].

**Abbreviated output (example format):**

- Boundary case: U14

Why: overlaps Theme 2 and Theme 4 / contradicts assumption of…

Suggestion: refine Theme 2 definition to include… or split…

**How the team used this output:**

- Used to refine theme boundaries and ensure that themes were not overly inclusive.

## **Optional: Cohort comparison prompt (bounded language)**

**Prompt:**
Compare Cohort 2021 vs 2022 descriptively based only on the coded summaries provided. Avoid causal language. Output: (1) 2–3 observed differences, (2) 1–2 plausible interpretations stated as hypotheses, (3) a sentence noting alternative explanations.
