# Supplementary material for: Impact of Community-Oriented Medical Education on Medical Students’ Perceptions of Community Health Care: Qualitative Study
Source: JMIR Med Educ. 2026 Jan 19;12:e84406. doi: 10.2196/84406 (PMC12865343; doi:10.2196/84406)
Supplement: Multimedia Appendix 4 [file mededu_v12i1e84406_app4.docx]

**Supplementary file 4. Results of qualitative content analysis of first-year regional quota medical students’ reflection reports in the 2021 Community-Oriented Medical Education (COME) program at Chiba University (n = 20).**

| **Fink’s Taxonomy of Significant Learning** | **Subcategory** | **Quote** |
| --- | --- | --- |
| Learning How to Learn (6) | Practical experience and activity participation (2) | *"Practical experience enhances learning."* |
|  | Improvement of skills and knowledge (2) | *"Continuous learning improves skills."* |
|  | Improvement of communication skills (1) | *"Providers should develop communication skills."* |
|  | Continuous learning and understanding of community (1) | *"Providers should engage in continuous learning."* |
| Caring (30) | Contribution and involvement in community healthcare (9) | *"Providers should contribute to community health."* |
|  | Personal growth and learning (8) | *"Personal growth is important for healthcare providers."* |
|  | Patient-centered healthcare (7) | *"Patient-centered care focuses on patients' needs."* |
|  | Communication and collaboration (5) | *"Effective communication is essential for collaboration."* |
|  | Personal motivation and awareness (1) | *"Personal motivation is important for providers."* |
| Human Dimension (16) | Patient-centered healthcare and communication (6) | *"Patient-centered care focuses on patient needs."* |
|  | Understanding and importance of community healthcare (5) | *"Understanding community healthcare is essential for providers."* |
|  | General practice and broad knowledge (3) | *"General practitioners should have broad medical knowledge."* |
|  | Realization of community healthcare field (2) | *"Community healthcare is a vital field."* |
| Integration (40) | Challenges and responses in community healthcare (13) | *"Addressing healthcare provider shortages is a major challenge."* |
|  | Improvement of knowledge and skills (11) | *"Continuous education is essential for healthcare providers."* |
|  | Communication and cooperation (9) | *"Effective communication is essential for healthcare delivery."* |
|  | Collaboration with the community (7) | *"Community collaboration improves healthcare delivery."* |
| Application (91) | Improvement of knowledge and skills (20) | *"Continuous education is essential for healthcare providers."* |
|  | Practice and problem-solving in community healthcare (18) | *"Problem-solving skills are essential for managing patient care effectively."* |
|  | Communication and trust building (15) | *"Effective communication is essential for building trust with patients."* |
|  | Necessity and outlook of community healthcare (13) | *"Community healthcare is necessary for addressing health disparities."* |
|  | Improvement of prevention and health awareness (9) | *"Health education programs are essential for improving health awareness."* |
|  | Utilization of information and communication technology (7) | *"Telemedicine services can improve access to healthcare in remote areas."* |
|  | Environmental preparation and collaboration in community healthcare (5) | *"Collaboration with environmental agencies is essential for community health."* |
|  | General practitioners and multi-disciplinary knowledge (4) | *"General practitioners should have a broad knowledge of different medical fields."* |
| Foundational Knowledge (128) | Specific initiatives in community healthcare (48) | *"Healthcare providers must be able to identify and address health problems in the community."* |
|  | Challenges and current state of community healthcare (38) | *"Community healthcare is essential for ensuring access to healthcare for all."* |
|  | Definition and role of community healthcare (26) | *"Community healthcare is important for health equity."* |
|  | Aging population and community healthcare (16) | *"Community healthcare is necessary for supporting aging populations."* |
| *() number of codes |  |  |

**Note:** Reports were analyzed using Fink’s Taxonomy of Significant Learning, which categorizes cognitive domains into six categories. Representative quotes from students’ reflections are shown for each subcategory to illustrate perceptions of community healthcare prior to the program revision.

**Table 2. Results of qualitative content analysis of first-year regional quota medical students’ reflection reports in the revised 2022 Community-Oriented Medical Education (COME) program at Chiba University (n = 20).**

| **Fink’s Taxonomy of Significant Learning** | **Subcategory** | **Quote** |
| --- | --- | --- |
| Learning How to Learn (15) | Acquisition of broad knowledge (5) | *"Acquiring a broad range of knowledge is important for comprehensive healthcare."* |
|  | Activities to broaden knowledge (4) | *"Engaging in activities that broaden knowledge can enhance healthcare delivery."* |
|  | Interest and training (3) | *"Ongoing interest and training in healthcare are essential for professional development."* |
|  | Mandatory community healthcare training (2) | *"Mandatory training in community healthcare ensures that providers are well-prepared."* |
|  | Improvement of communication skills (1) | *"Improving communication skills is critical for effective patient interactions."* |
| Caring (58) | Communication and collaboration (15) | *"Collaboration among healthcare providers enhances communication and patient care."* |
|  | Contribution and involvement in community healthcare (12) | *"Active involvement in community healthcare initiatives is crucial for success."* |
|  | Patient-centered healthcare (11) | *"Patient-centered healthcare ensures that the care provided meets the needs of the patient."* |
|  | Personal growth and learning (10) | *"Healthcare providers should focus on personal growth and continuous learning."* |
|  | Personal motivation and awareness (10) | *"Personal motivation and awareness are key drivers for effective healthcare delivery."* |
| Human Dimension (57) | Patient-centered healthcare and communication (26) | *"Patient-centered healthcare prioritizes effective communication with patients."* |
|  | Challenges and improvements in community healthcare (21) | *"Addressing the challenges in community healthcare requires continuous improvement efforts."* |
|  | Importance and current state of community healthcare (10) | *"Understanding the current state of community healthcare is essential for making improvements."* |
| Integration (45) | Medical resources and collaboration (15) | *"Collaboration between medical institutions is essential for optimizing resources."* |
|  | Sustainability and technology in healthcare (12) | *"Sustainable healthcare practices and modern technology can improve patient outcomes."* |
|  | Patient-centered care method (10) | *"Patient-centered care focuses on the individual needs and preferences of patients."* |
|  | Ethics and communication (8) | *"Ethical considerations are paramount in healthcare communication and decision-making."* |
| Application (82) | Improvement of medical knowledge and skills (20) | *"Continuous education and training are essential for improving medical knowledge and skills."* |
|  | Practice and problem-solving in community healthcare (20) | *"Healthcare providers must be adept at problem-solving to address diverse community health issues."* |
|  | Communication and trust building (15) | *"Building trust through clear and compassionate communication is key in healthcare."* |
|  | Community healthcare policy and support (8) | *"Effective policies and support systems are necessary for sustaining community healthcare."* |
|  | Executing ability and motivation (8) | *"Healthcare providers need both the ability and motivation to execute their duties effectively."* |
|  | Importance of community healthcare (6) | *"Community healthcare plays a vital role in ensuring equitable access to medical services."* |
|  | Utilization of information and communication technology (5) | *"ICT can enhance healthcare delivery, especially in remote areas."* |
| Foundational Knowledge (128） | Quality and supply of healthcare (51) | *"Ensuring high-quality healthcare is critical for meeting community needs."* |
|  | Shortage of physicians and medical resources (41) | *"Many rural areas face significant shortages of doctors and medical resources."* |
|  | Importance of communication and consultation (23) | *"Effective communication between healthcare providers and patients is crucial for accurate diagnosis and treatment."* |
|  | Aging population and community healthcare (13) | *"The aging population increases the demand for community healthcare services."* |
| *() number of codes |  |  |

**Note:** The revised program included an additional early clinical exposure component at hospitals staffed by COME-trained attending physicians. Data were analyzed using Fink’s Taxonomy of Significant Learning, and representative participant quotes are provided for each subcategory to highlight changes in students’ understanding after program enhancement.
