# Supplementary material for: Impact of Community-Oriented Medical Education on Medical Students’ Perceptions of Community Health Care: Qualitative Study
Source: JMIR Med Educ. 2026 Jan 19;12:e84406. doi: 10.2196/84406 (PMC12865343; doi:10.2196/84406)
Supplement: Multimedia Appendix 5 [file mededu_v12i1e84406_app5.docx]

**Supplementary file 5. Results of qualitative content analysis of first-year regional quota medical students’ reflection reports in the revised 2022 Community-Oriented Medical Education (COME) program at Chiba University (n = 20).**

| **Fink’s Taxonomy of Significant Learning** | **Subcategory** | **Quote** |
| --- | --- | --- |
| Learning How to Learn (15) | Acquisition of broad knowledge (5) | *"Acquiring a broad range of knowledge is important for comprehensive healthcare."* |
|  | Activities to broaden knowledge (4) | *"Engaging in activities that broaden knowledge can enhance healthcare delivery."* |
|  | Interest and training (3) | *"Ongoing interest and training in healthcare are essential for professional development."* |
|  | Mandatory community healthcare training (2) | *"Mandatory training in community healthcare ensures that providers are well-prepared."* |
|  | Improvement of communication skills (1) | *"Improving communication skills is critical for effective patient interactions."* |
| Caring (58) | Communication and collaboration (15) | *"Collaboration among healthcare providers enhances communication and patient care."* |
|  | Contribution and involvement in community healthcare (12) | *"Active involvement in community healthcare initiatives is crucial for success."* |
|  | Patient-centered healthcare (11) | *"Patient-centered healthcare ensures that the care provided meets the needs of the patient."* |
|  | Personal growth and learning (10) | *"Healthcare providers should focus on personal growth and continuous learning."* |
|  | Personal motivation and awareness (10) | *"Personal motivation and awareness are key drivers for effective healthcare delivery."* |
| Human Dimension (57) | Patient-centered healthcare and communication (26) | *"Patient-centered healthcare prioritizes effective communication with patients."* |
|  | Challenges and improvements in community healthcare (21) | *"Addressing the challenges in community healthcare requires continuous improvement efforts."* |
|  | Importance and current state of community healthcare (10) | *"Understanding the current state of community healthcare is essential for making improvements."* |
| Integration (45) | Medical resources and collaboration (15) | *"Collaboration between medical institutions is essential for optimizing resources."* |
|  | Sustainability and technology in healthcare (12) | *"Sustainable healthcare practices and modern technology can improve patient outcomes."* |
|  | Patient-centered care method (10) | *"Patient-centered care focuses on the individual needs and preferences of patients."* |
|  | Ethics and communication (8) | *"Ethical considerations are paramount in healthcare communication and decision-making."* |
| Application (82) | Improvement of medical knowledge and skills (20) | *"Continuous education and training are essential for improving medical knowledge and skills."* |
|  | Practice and problem-solving in community healthcare (20) | *"Healthcare providers must be adept at problem-solving to address diverse community health issues."* |
|  | Communication and trust building (15) | *"Building trust through clear and compassionate communication is key in healthcare."* |
|  | Community healthcare policy and support (8) | *"Effective policies and support systems are necessary for sustaining community healthcare."* |
|  | Executing ability and motivation (8) | *"Healthcare providers need both the ability and motivation to execute their duties effectively."* |
|  | Importance of community healthcare (6) | *"Community healthcare plays a vital role in ensuring equitable access to medical services."* |
|  | Utilization of information and communication technology (5) | *"ICT can enhance healthcare delivery, especially in remote areas."* |
| Foundational Knowledge (128） | Quality and supply of healthcare (51) | *"Ensuring high-quality healthcare is critical for meeting community needs."* |
|  | Shortage of physicians and medical resources (41) | *"Many rural areas face significant shortages of doctors and medical resources."* |
|  | Importance of communication and consultation (23) | *"Effective communication between healthcare providers and patients is crucial for accurate diagnosis and treatment."* |
|  | Aging population and community healthcare (13) | *"The aging population increases the demand for community healthcare services."* |
| *() number of codes |  |  |

**Note:** The revised program included an additional early clinical exposure component at hospitals staffed by COME-trained attending physicians. Data were analyzed using Fink’s Taxonomy of Significant Learning, and representative participant quotes are provided for each subcategory to highlight changes in students’ understanding after program enhancement.
